# Comparative Autonomic Responses to Diagnostic Interviewing between Individuals with GAD, MDD, SAD and Healthy Controls

**DOI:** 10.3389/fnhum.2016.00677

**Published:** 2017-01-11

**Authors:** Allison E. Diamond, Aaron J. Fisher

**Affiliations:** Idiographic Dynamic Laboratory, Department of Psychology, University of California, BerkeleyBerkeley, CA, USA

**Keywords:** clinical interview, perseverative cognition, autonomic nervous system, generalized anxiety disorder, major depressive disorder

## Abstract

Dysregulation of the autonomic nervous system (ANS) has been well documented in individuals diagnosed with a range of psychological disorders, including generalized anxiety disorder (GAD) and major depressive disorder (MDD). Moreover, these disorders both confer an increased risk of cardiovascular disease—which may relate to increased sympathetic and decreased parasympathetic tone. Extant research has indicated a reduction in autonomic flexibility in GAD, and while reduced flexibility has also been seen in MDD, the specific physiological alterations have been more difficult to categorize due to methodological limitations, including high co-morbidity rates with anxiety disorders. Prior studies have largely assessed autonomic functioning in stress paradigms or at the trait level, yet to date, no research has investigated the ANS during a diagnostic interview, a ubiquitous task employed in both research and clinical settings. In this study we sought to identify physiological differences in both branches of the ANS across diagnostic categories in the context of a diagnostic interview. Participants (*n* = 82) were administered a structured clinical interview, during which heart rate (HR), respiratory sinus arrhythmia (RSA) and pre-ejection period (PEP) were recorded in participants carrying a diagnosis of GAD (*n* = 34), MDD (*n* = 22), Social Anxiety Disorder (SAD; *n* = 15) and healthy controls (*n* = 27). Person-specific linear regression models were employed to assess the level and slope for HR, RSA and PEP throughout the course of the interview. A multivariate analysis of variance (MANOVA) model was conducted to baseline differences in HR, RSA and PEP between diagnostic groups. Multiple regression models were then conducted to differences in slope of HR, RSA and PEP throughout the course of the interview amongst diagnostic groups, including both suppression and worry as moderators. Results indicated significant increases in RSA throughout the interview in MDD (*p* = 0.01) compared to healthy controls. Worry itself was found to be a more significant predictor of both decreased PEP (*p* = 0.02) and increased HR (*p* = 0.05). Suppression exhibited a dampening effect on individuals with worry and GAD, whereby those who suppressed had dampened HR responsiveness compared to those who did not suppress. These findings are consistent with existing literature supporting a decreased autonomic flexibility in certain psychological disorders, as well as indicate distinct physiological differences across certain transdiagnostic features of mood and anxiety disorders.

## Introduction

The autonomic nervous system (ANS) is a principal driver of physiologic regulation (Berntson and Cacioppo, 2007), helping to facilitate adaptive responses to environmental demands. Under even moderate stress conditions such as mental arithmetic (Sloan et al., 1991) or social stress tasks (Nater et al., 2006; Hellhammer and Schubert, 2012), inhibitory signals from the parasympathetic nervous system (PNS) are typically downregulated and sympathetic nervous system (SNS) arousal upregulated as a part of an adaptive stress response. Research has consistently shown, however, that individuals with generalized anxiety disorder (GAD) exhibit reduced autonomic flexibility (Hoehn-Saric and McLeod, 2000)—muted PNS and SNS responses to experimental and ecological stress (Thayer et al., 1996; Hoehn-Saric et al., 2004; Fisher et al., 2010; Fisher and Newman, 2016). Rigid or inflexible ANS responsiveness may inhibit adaptive responses to environmental demands, and autonomic rigidity has been considered an indicator of poor health and has been associated with increased susceptibility to cardiovascular disease (Thayer and Lane, 2007). Autonomic inflexibility has also been seen in major depressive disorder (MDD; Udupa et al., 2007; Koschke et al., 2009), although evidence has suggested that reduced PNS activity in MDD is a result of high comorbidity rates with anxiety disorders, rather than an effect of depression itself (Friedman, 2007; Rottenberg, 2007). Nevertheless, there is evidence that individuals with mood and anxiety diagnoses exhibit regulatory impairments in autonomic functioning.

Although the traditional doctrine of autonomic reciprocity has heuristic value in understanding the counteracting up-regulation of SNS activity and down-regulation of PNS activity during stress, Berntson et al. (1991) have demonstrated that the PNS and SNS exist on independent axes. Thus, researchers investigating stress responsiveness should capture concurrent measurements of parasympathetic and sympathetic indices in order to investigate potential patterns of activation. As a target organ that is dually-innervated by PNS and SNS efferents and easily captured via cardiographic methods, the heart is an ideal and widely measured basis for assessing the concurrent influences of the SNS and PNS. The primary source of increased cardiovascular reactivity during stress is drawn from increased SNS activity (Hjemdahl et al., 1989), as the SNS predominantly regulates changes in heart rate (HR) during periods of increased metabolic demand. A measure of cardiac sympathetic control, cardiac pre-ejection period (PEP), can be continuously acquired through thoracic impedance cardiography (ICG). PEP is defined as the period between electrical invasion of the ventricular myocardium (Q wave of the ECG) and the opening of the aortic valve, and is obtained by measuring the period of time between depolarization of the left ventricle of the heart and the onset of ejection of the blood into the aorta. Shorter PEP reflects increased sympathetic activation. Conversely, parasympathetic regulatory influences predominate cardiac control at rest. These signals are inferred via the calculation of respiratory sinus arrhythmia (RSA) from the interbeat intervals of the ECG signal. RSA is the fluctuation of heart period related to respiratory oscillations, and is used as a proxy measurement for parasympathetic control, given that vagal signaling cannot be directly measured or observed in humans.

Investigations of autonomic regulation in mood and anxiety populations have employed a number of methods for stress induction, including the Trier social stress task (Hellhammer and Schubert, 2012), mental arithmetic and other performative challenges (Fisher and Newman, 2013), emotionally-evocative images and video (Fisher et al., 2010), as well inductions of worry and perseverative cognition (Borkovec and Hu, 1990). Defined by Brosschot et al. (2006) as the repeated or chronic activation of the cognitive representation of one or more physiological stressors, perseverative cognition encompasses elements of both worrisome thinking and rumination, putative cardinal features of both GAD and MDD, respectively. Utilizing this broader terminology, researchers have shown that perseverative cognition is associated with lower levels of cognitive flexibility and increased autonomic rigidity (Ottaviani et al., 2013, 2016), elucidating a possible connection between cognitive processes and ANS dysfunctions.

Worry itself, regardless of diagnosis, has also been related to diminished stress reactivity (see Borkovec and Hu, 1990). Worrisome thinking prior to exposure to phobic imagery has been shown to inhibit cardiovascular response in anxious individuals (Borkovec and Hu, 1990; Borkovec et al., 1993; Llera and Newman, 2010) and, in a laboratory setting, worry inductions have been shown to lead to dampened RSA in both anxious and non-anxious individuals (Lyonfields et al., 1995; Thayer et al., 1996). Furthermore, in healthy adults, experimentally-induced worry has been shown to predict higher HR and lower RSA when compared to a non-worry resting baseline (Verkuil et al., 2009), and lastly, in an ambulatory study of healthy adults, worry consistently predicted higher HR and lower RSA during waking, and the duration of worry significantly predicted lower RSA in both waking and sleeping conditions (Brosschot et al., 2007). Thus, there is strong evidence to suggest a potential causal relationship between worrisome thinking and diminished ANS responsiveness.

Brosschot et al.’s 2006 perseverative cognition hypothesis presents a coherent framework for understanding the association between cognitive representations of stressful events and ANS changes. Consistent with Lazarus’ paradigm-shifting proposal that stress reactions can result from purely psychological representations of threat (see Lazarus and Folkman, 1984), the perseverative cognition hypothesis posits that stress reactions can occur regardless of a stressor’s presence. Thus, individuals can form mental representations of stressful events both before and after the events are meant to happen, even if the events do not actually occur. In turn, normative physiologic stress responses can result from these cognitive representations. A number of theoretical and empirical observations have supported this hypothesis (Borkovec and Hu, 1990; Lyonfields et al., 1995; Thayer et al., 1996; Aldao et al., 2014).

Perseverative cognition as an emotion regulation strategy has been posited as a vehicle of experiential avoidance (Borkovec et al., 2004; Roemer et al., 2005; Tull et al., 2011). In particular, experiential avoidance theories of worry in GAD propose that because individuals with GAD find emotionally-evocative experiences threatening, worry is employed to suppress negative emotionality (Mennin et al., 2005), provide distraction from emotional topics (Borkovec and Roemer, 1995) and preclude the emotional processing of fearful stimuli (Borkovec et al., 2004). Perhaps foremost of these theories is Borkovec’s Avoidance Model of Worry (Borkovec, 1994; Borkovec et al., 2004), which proposes that worry is a verbal-linguistic cognitive process that prevents engagement with more emotionally-evocative mental imagery and thus distracts worriers from more emotional topics (Borkovec and Roemer, 1995).

Physiologic data support the theory that worry suppresses engagement with—or at least the somatic experience of—negative emotions, as stated previously. Moreover, this effect has also been extended outside of GAD. Speech-anxious individuals who engaged in worrisome thinking prior to a public speaking exposure showed lesser cardiovascular reactivity compared to those who did not worry prior to exposure (Borkovec and Hu, 1990), and levels of self-reported worry across healthy control and GAD participants negatively predicted the degree of HR reactivity to a laboratory stress paradigm (Fisher and Newman, 2013). Thus, there is consistent support for the notion that perseverative cognition suppresses physiologic reactions to stress. Yet, there is also evidence that individuals with GAD, MDD and social anxiety disorder (SAD; Amstadter, 2008; Spokas et al., 2009; Beblo et al., 2012) engage in emotion regulation strategies beyond perseverative cognition, including emotional suppression, an overt and specific form of emotional avoidance that involves the conscious inhibition of emotional experiences during emotionally-evocative events or experiences (Gross and Levenson, 1993). Suppression is often characterized as ineffective and maladaptive, with associated deleterious effects such as memory impairment (Richards and Gross, 1999, 2000), reduced cognitive abilities (Richards and Gross, 1999) and decreased emotionally-expressive behavior. Consistent with experiential avoidance, emotional suppression has been shown to result in attenuated HR reactivity for fear and disgust (Gross and Levenson, 1993; Gross, 1998; Sloan, 2004; Reynaud et al., 2012).

Several research paradigms have attempted to leverage the Lazarus model of stress to investigate the physiologic stress response by inducing or invoking stress-related cognitive representations through worry (Fisher and Newman, 2013) or other imaginal procedures (Ottaviani et al., 2015). Yet, a compelling and relevant area that has received little empirical attention is the clinical diagnostic interview. During clinical interviews such as the Structured Clinical Interview for the DSM (SCID; First, 1995) and Anxiety and Related Disorders Interview Schedule (ADIS; Brown and Barlow, 2014), interviewers ask interviewees to bring to mind thoughts, feelings and experiences that reflect potential underlying psychopathology. For those who meet clinical criteria, such pathology is inherently distressing—a condition required by the Diagnostic and Statistical Manual of Mental Disorders, fifth Edition (DSM-5) to meet diagnostic criteria (American Psychiatric Association, 2013). Anecdotally, both researchers and clinicians have presupposed that clinical diagnostic interviews are stressful to interviewees (Lichstein, 1990), however; to our knowledge no one has examined this empirically to date.

The goals of the present study were to examine the potentially stressful nature of clinical diagnostic interviews in order to assess the degree to which these interviews induce physiologic stress reactions. Due to the high comorbidity rates among mood and anxiety disorders, we assessed for the presence vs. absence of three relevant diagnoses—GAD, MDD and SAD—allowing for overlapping co-occurrence, rather than investigate primary diagnoses. Thus, we were interested in distinguishing the shared and unique contributions of these diagnoses on ANS functioning. Given the robust research literature on diminished physiological flexibility in GAD, we were primarily interested in examining the relative reactions in individuals with a diagnosis of GAD compared to healthy controls, however we included individuals with MDD and SAD in order to further isolate the potentially unique contributions of GAD pathology to diminished physiological flexibility, when compared to diagnostically and phenomenologically similar diagnoses. Additionally, because worry has been specifically implicated as a suppressant of autonomic reactivity, we were interested in the degree to which *worry itself*, regardless of diagnosis, would possibly affect observable ANS responsiveness. Finally, due to previous findings of attenuated HR reactivity with emotional suppression, we were interested in examining the additional contribution of emotion suppression to the phenomenology and physiologic reactivity of individuals with GAD, MDD, SAD and healthy controls.

Individuals with principal diagnoses of GAD, MDD, SAD and healthy controls completed the ADIS-5 semi-structured clinical interview. During this interview electrocardiography (ECG) and ICG were measured in order to obtain HR, RSA and PEP. We examined the effect of: (1) the presence of any psychological diagnosis vs. healthy controls; (2) the presence of specific GAD, MDD and SAD diagnosis; (3) the effect of worry; and (4) the effect of suppression on trajectories of HR, RSA and PEP across the interview. The present study represents an exploratory examination of the degree to which clinical diagnostic interviews engage with and map onto psychological representations of stressful experiences and the potential effects of transdiagnostic features (i.e., worry, suppression) on such experiences.

## Materials and Methods

### Participants

The sample was composed of 82 participants: 24 individuals with a primary diagnosis of GAD, 18 individuals with a primary diagnosis of MDD, 13 individuals with a primary diagnosis of SAD and 27 healthy controls who did not meet criteria for any DSM-5 diagnoses. Table 1 provides participant characteristics by group. Of the 82 participants, 44% were Caucasian (*n* = 36), 21% were Asian American (*n* = 17), 17% were Latino (*n* = 14), 9% were African American (*n* = 7), 4% were Native American (*n* = 3) and 6% designated their cultural background as “other” (*n* = 5). Twenty-two of the participants who met criteria for a clinical disorder also met diagnostic criteria for at least one comorbid Axis I disorder (GAD, *n* = 10; MDD, *n* = 4; SAD, *n* = 3; bipolar disorder, *n* = 2; agoraphobia, *n* = 1; panic disorder, *n* = 1; posttraumatic stress disorder, *n* = 1), resulting in a sample of 34 individuals carrying a diagnosis of GAD, 22 individuals carrying a diagnosis of MDD and 15 individuals carrying a diagnosis of SAD. Participants were recruited by the use of flyers and craigslist advertisements, and underwent a phone screening before being included in the study. Exclusion criteria were: not being between the ages of 18 and 65, not having English proficiency (both written and spoken), not being able to commute to the UC Berkeley Campus, not having regular access to a mobile phone that receives text messages, has internet access, and has a touchscreen, and having current CBT or having had CBT in the past year. All participants consented to the study, and were compensated for their time ($35). IRB approval was obtained by the University of California, Berkeley Institutional Review Board. Verbal consent was obtained during the first phone screening to rule out exclusionary criterion. Written consent was obtained upon the first lab visit, prior to any experimental procedures.

**Table 1:** **Participant characteristics by principal diagnosis**.

	Control (*n* = 27)	GAD (*n* = 24)	MDD (*n* = 18)	SAD (*n* = 13)
Age	Mean: 34.46	Mean: 32.50	Mean: 35.28	Mean: 26.38
	*SD*: 14.59	*SD*: 12.11	*SD*: 14.98	*SD*: 10.06
Sex	Male: *n* = 10 (37%)	Male: *n* = 8 (33%)	Male: *n* = 7 (39%)	Male: *n* = 3 (23%)
	Female: *n* = 17 (63%)	Female: *n* = 16 (67%)	Female: *n* = 11 (61%)	Female: *n* = 10 (77%)
Education	Median = 4	Median = 4	Median = 4	Median = 4
BMI	Mean = 24.81	Mean = 24.81	Mean = 27.24	Mean = 22.85
	SD = 4.91	SD = 5.82	SD = 13.48	SD = 6.90

*Note: GAD, Generalized Anxiety Disorder; MDD, Major Depressive Disorder; SAD, Social Anxiety Disorder; BMI, body mass index. Education coded as: 3 = “some college”, 4 = “4 year college degree”*.

### Procedure

After a pre-screening phone interview, participants came into the lab, read and signed the informed consent form, and completed a series of questionnaires covering psychopathological constructs and socio-demographics (biological sex, age, income, race, ethnicity, religion, marital status, occupational history and education). Following this, participants were outfitted with electrodes for measurement of peripheral physiology, (see below), and completed the Anxiety Disorders Interview Schedule (ADIS-IV; Brown et al., 1994), administered by a graduate student or post-graduate research assistant. The ADIS-IV is a semi-structured interview developed to establish differential diagnoses of anxiety and mood disorders, and has excellent retest reliability and high interrater reliability (Brown et al., 2001). Assessors also completed the Hamilton Anxiety Rating Scale (HARS; Hamilton, 1959), Hamilton Rating Scale for Depression (HRSD; Hamilton, 1960) and CSRs for GAD and comorbid disorders. Following the interview, participants received monetary compensation.

### Measures

Self-report measures collected prior to diagnostic interview included the following: the emotion regulation questionnaire and Penn State worry questionnaire.

#### Emotion Regulation Questionnaire (ERQ10; Gross and John, 2003)

The ERQ10 measures participants’ tendency to regulate their emotions in two ways: (1) cognitive reappraisal; and (2) expressive suppression. Participants respond to each item on a 7-point likert scale ranging from 1 (strongly disagree) to 7 (strongly agree). Previous analyses on the ERQ10 have indicated alpha reliabilities of 0.79 for reappraisal and 0.73 for suppression, and test-retest reliability across 3 months was 0.69 for both scales (Gross and John, 2003).

#### Penn State Worry Questionnaire (PSWQ; Meyer et al., 1990)

The PSWQ is a 16 item self-report measure of pathological worry. Factor analysis indicated that the PSWQ assesses a unidimensional construct with internal consistency of 0.91 (Meyer et al., 1990). High retest reliability (ranging from 0.74 to 0.93) has also been demonstrated across periods ranging from 2 to 10 weeks (Molina and Borkovec, 1994).

### Physiological Recordings

HR and RSA data acquisition followed standard guidelines (Berntson et al., 1997) using a Bioamp data acquisition system (MindWare Technologies, Inc., Gahanna, OH, USA). Disposable silver/silver-chloride (Ag-AgCl) electrodes were placed on participants. For the electrocardiogram (ECG), electrodes were placed on participant’s right collarbone and 10th-left and right ribs. For ICG, two voltage electrodes were placed below the suprasternal notch and xiphoid process, and two current electrodes were placed on the back 3–4 cm above and below the voltage electrodes. All electrodes were placed by a same-sex RA. ECG and ICG were recorded throughout the duration of the clinical interview at a sampling rate of 500 Hz.

RSA was scored and quantified by extracting the high frequency spectral component of the R-R peak time series (0.15–0.40 Hz). Artifacts were detected and corrected manually using standard procedures (Berntson et al., 1997). RSA was then derived using spectral analysis (Berntson et al., 1997), using 30-s epochs.

Cardiac PEP was derived from the ECG and ICG in 30 s epochs, using MindWare ICG V. 2.3. PEP was indexed as the time interval in milliseconds from the onset of the Q-wave to the B point of the dZ/dt wave, using validated methods (Berntson et al., 2004). Using the software, artifacts were examined and removed via visual inspection.

### Approach to Statistical Analyses

Linear trajectories for changes in HR, RSA and PEP during diagnostic interviewing were assessed on a person by person basis via ordinary least squares regression. A regression model was conducted for each individual such that time-varying HR, RSA and PEP values were regressed on time (epoch number). The standardized regression coefficients were then extracted and aggregated into a standard nomothetic data set in order to assess individual differences in slopes per study hypotheses.

To evaluate the effects of diagnosis on baseline HR, RSA and PEP, a multivariate analysis of variance (MANOVA) was conducted for differences in HR, RSA and PEP during the introductory module of the ADIS-5, while controlling for sex and age.

To investigate the effect of clinical diagnoses on physiologic stress responses to the diagnostic interview, three multiple regression models were conducted for HR, RSA and PEP, respectively, with GAD, MDD and SAD as predictors. We covaried age, sex and baseline levels of the dependent variable as control variables. To examine the moderating role of suppression in GAD, MDD and SAD, for each model we tested the moderating role of suppression via an interaction term between diagnosis and the ERQ suppression subscale.

To examine the role of worry on physiological functioning in GAD, MDD and SAD, three multiple regression models were conducted for HR, RSA and PEP respectively, with worry as a predictor. Worry was defined as PSWQ score. We covaried age, sex and baseline levels of the dependent variable as control variables. Again, to examine the role of emotional suppression on worry, for each model we tested the moderating role of suppression via an interaction term between diagnosis and the ERQ suppression subscale. All statistical analyses were performed in R 2.1 (R Core Team, 2015).

## Results

### Preliminary Analysis of Baseline Differences

In order to examine baseline differences in autonomic arousal across individuals with GAD, SAD, MDD and healthy controls, a MANOVA was conducted for differences in HR, RSA and PEP during the introductory module of the ADIS-5, while controlling for sex and age. Results of the MANOVA revealed that age (*F*_(3,67)_ = 17.77, *p* < 0.001) was a significant predictor of baseline differences with no significant effects for sex (*F*_(3,67)_ = 0.50, *p* = 0.69), GAD (*F*_(3,67)_ = 1.04, *p* = 0.38), MDD (*F*_(3,67)_ = 1.18, *p* = 0.32) or SAD (*F*_(3,67)_ = 1.73, *p* = 0.17). *Post hoc* univariate analyses revealed that age was a significant, positive predictor of RSA at baseline (*F*_(5,76)_ = 8.35, *p* < 0.001).

### Assessing Differences Amongst Clinical Groups vs. Healthy Controls on the Moderating Role of Suppression in HR, RSA and PEP across Diagnostic Interview

In order to investigate the presence of physiologic stress responses to the diagnostic interview, three multiple regression models were conducted for RSA, PEP and HR trajectories, respectively. For each model, in addition to testing the main effects for group differences between healthy controls and those with the presence of GAD, MDD and SAD diagnoses, we covaried age, sex and baseline levels of the dependent variable as control variables. Given our hypothesis related to the role of emotional suppression, for each model we tested the moderating role of suppression via an interaction term between GAD, MDD and SAD and the ERQ suppression subscale. Results for both the main effect and interaction models are presented in Table 2.

**Table 2:** **Regression model for interactions between suppression and clinical diagnosis on physiological responses**.

	RSA					PEP					HR				
	beta	SE	*t*	*p*	*d*	beta	SE	*t*	*p*	*d*	beta	SE	*t*	*p*	*d*
**Main effect model (*R*^2^ = 0.31; *R*^2^ = 0.26; *R*^2^ = 0.19)**															
Intercept	−0.41	0.19	−2.12	0.04	−0.58	0.39	0.20	1.98	0.05	0.54	−0.23	0.19	−1.12	0.23	−0.30
GAD	0.26	0.22	1.18	0.24	0.29	−0.47	0.23	−2.03	0.05	−0.49	0.27	0.22	1.23	0.22	0.30
SAD	0.32	0.31	1.05	0.30	0.38	−0.41	0.34	−1.23	0.22	−0.45	0.32	0.31	1.05	0.23	0.38
MDD	0.68	0.25	2.65	0.01	0.80	−0.22	0.27	−0.82	0.42	−0.25	−0.04	0.25	−0.14	0.89	−0.04
Suppression	−0.23	0.11	−2.04	0.04	−0.32	−0.13	0.12	−1.06	0.23	−0.17	0.15	0.12	1.27	0.21	0.20
Sex (male)	−0.01	0.24	−0.03	0.98	−0.01	−0.11	0.25	−0.46	0.65	−0.10	0.21	0.24	0.90	0.37	0.20
Age	0.02	0.13	0.14	0.89	0.02	0.05	0.12	0.46	0.65	0.07	−0.08	0.11	−0.68	0.50	−0.11
Baseline	−0.36	0.13	−2.82	0.01	−0.44	−0.38	0.12	−3.15	<0.001	−0.49	−0.27	0.11	−2.36	0.02	−0.30
**Interaction model (*R*^2^ = 0.38; *R*^2^ = 0.25; *R*^2^ = 0.30)**															
Intercept	−0.27	0.20	−1.36	0.18	−0.37	0.42	0.20	2.07	0.04	0.56	−0.38	0.20	−1.96	0.05	−0.53
GAD	0.17	0.22	0.74	0.46	0.18	−0.52	0.24	−2.12	0.04	−0.51	0.37	0.22	1.37	0.10	0.33
SAD	0.34	0.32	1.06	0.30	0.39	−0.34	0.37	−0.93	0.35	−0.34	0.31	0.31	1.00	0.32	0.37
MDD	0.41	0.27	1.52	0.14	0.46	−0.25	0.28	−0.89	0.38	−0.27	0.24	0.26	0.91	0.37	0.27
Suppression	−0.67	0.21	−3.19	<0.001	−0.50	−0.27	0.20	−1.30	0.20	−0.20	0.63	0.20	3.12	<0.001	0.49
Sex (male)	0.12	0.24	0.52	0.61	0.11	−0.07	0.25	−0.26	0.79	−0.06	0.06	0.23	0.28	0.78	0.06
Age	0.06	0.13	0.45	0.65	0.07	0.06	0.12	0.47	0.64	0.07	−0.08	0.11	−0.74	0.46	−0.12
Baseline	−0.29	0.13	−2.32	0.02	−0.36	−0.36	0.12	−3.07	<0.001	−0.48	−0.26	0.11	−2.32	0.02	−0.36
GAD × Supp.	0.51	0.24	2.15	0.04	0.52	0.04	0.25	0.16	0.87	0.04	−0.57	0.22	−2.55	0.01	−0.62
SAD × Supp.	0.54	0.36	1.50	0.14	0.55	0.36	0.40	0.91	0.37	0.33	−0.57	0.35	−1.61	0.11	−0.59
MDD × Supp.	0.28	0.27	1.04	0.30	0.31	0.29	0.29	0.10	0.32	0.03	−0.30	0.26	−1.15	0.26	−0.35

Note: Reference group = healthy controls; GAD, Generalized Anxiety Disorder; MDD, Major Depressive Disorder; SAD, Social Anxiety Disorder; d = Cohen’s d (calculated as d = t * sqrt[2/n]).

Healthy controls exhibited a significant decrease in RSA throughout the course of the interview. Relative to healthy controls, individuals with MDD exhibited a significant increase in RSA throughout the course of the interview. Coefficients for RSA slope for individuals with GAD and SAD did not significantly differ from health controls. Suppression exhibited a significant main effect, such that higher levels of suppression predicted reductions in RSA across the interview in all participants. Finally, baseline levels of RSA were a significant predictor of RSA slope such that higher levels of RSA at baseline predicted decreased RSA across the interview.

**Figure 1:**
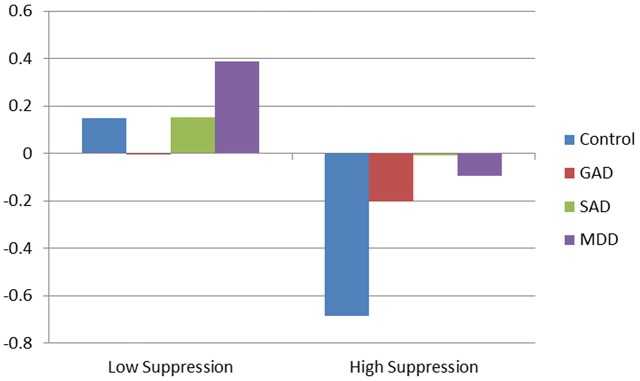
**Effect of clinical diagnosis on respiratory sinus arrhythmia (RSA) during diagnostic interview**.

The addition of interactions between suppression and diagnoses explained an additional 7% of the variance, compared to the main effect model (*R*^2^ = 0.31, *R*^2^ = 0.38, respectively). For healthy controls, those who suppressed exhibited significantly decreased RSA compared to those who did not suppress. Additionally, the interaction between GAD and suppression indicated that individuals with GAD who suppressed had significantly dampened RSA responses compared to healthy controls. No significant moderating effect of suppression was found for MDD or SAD participants. Results of RSA trajectory by group, as a function of emotional suppression, are depicted in Figure 1.

Results for PEP indicated that baseline PEP was a significant predictor of PEP slope in both the main effect and interaction models. No other significant predictors were found in the main effect model. However, given small cell sizes among the subgroups under analysis, it is prudent to point to medium effect sizes for healthy controls and individuals with GAD and SAD. These effects reveal that healthy controls appeared to show an increase in PEP across the interview (reflecting a decrease in SNS arousal) and GAD and SAD participants appeared to exhibit relative decreases in PEP (reflecting relative increases in SNS arousal). No moderating effects were found in the interaction model.

**Figure 2:**
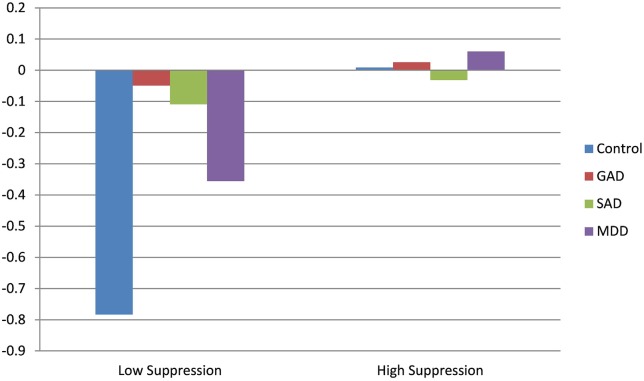
**Effect of clinical diagnosis on heart rate (HR) throughout the course of the diagnostic interview**.

Results for the main effect model for changes in HR during the diagnostic interview indicated only a significant effect for baseline HR. Consistent with both RSA and PEP, higher HR at baseline predicted decreases in HR across the interview. However, the addition of interactions between diagnosis and suppression revealed a significant effect of suppression on HR trajectory in healthy controls, such that higher suppression predicted significant increases in HR during the interview. Conversely to this, individuals with GAD with higher levels of suppression exhibited significantly flattened (i.e., muted) HR responses, compared to controls and GAD participants with low levels of suppression. Results of HR trajectory by group, as a function of emotional suppression, are depicted in Figure 2.

### Assessing Differences in Levels of Worry on the Moderating Role of Suppression in HR, RSA and PEP across Diagnostic Interview

In order to assess the role of worry as a predictor of autonomic responses across the diagnostic interview, each of the analyses reported above were repeated with the dimensional construct of worry severity inserted in the place of clinical diagnosis. We once again covaried age, sex and baseline levels of the dependent variable as control variables. For each model we tested the moderating role of suppression via an interaction term between the PSWQ score and the ERQ suppression subscale. Results for both the main effect and interaction models are depicted in Table 3.

**Table 3:** **Regression model for interactions between suppression and worry on physiological responses**.

	RSA					PEP					HR				
	beta	SE	*t*	*p*	*d*	beta	SE	*t*	*p*	*d*	beta	SE	*t*	*p*	*d*
**Main effect model (*R*^2^ = 0.22; *R*^2^ = 0.31; *R*^2^ = 0.23)**															
Intercept	−0.10	0.14	−0.74	0.47	−0.12	0.11	0.13	0.86	0.40	0.13	−0.11	0.13	−0.88	0.38	−0.14
Worry	0.19	0.13	1.50	0.14	0.23	−0.42	0.12	−3.64	<0.001	−0.57	0.27	0.12	2.27	0.03	0.35
Suppression	−0.24	0.12	−1.97	0.05	−0.31	−0.16	0.12	−1.35	0.18	−0.21	0.17	0.11	1.51	0.14	0.24
Sex (male)	0.16	0.26	0.63	0.53	0.14	−0.29	0.24	−1.21	0.23	−0.27	0.27	0.24	1.14	0.26	0.25
Age	0.09	0.14	0.66	0.51	0.10	0.01	0.12	0.08	0.94	0.01	−0.07	0.11	−0.62	0.54	−0.10
Baseline	−0.29	0.14	−2.06	0.04	−0.32	−0.40	0.11	−3.52	<0.001	−0.55	−0.24	0.11	−2.23	0.03	−0.35
**Interaction model (*R*^2^ = 0.32, *R*^2^ = 0.32, *R*^2^ = 0.32)**															
Intercept	0.00	0.13	0.01	0.99	0.00	0.13	0.13	0.98	0.33	0.15	−0.20	0.12	−1.65	0.10	−0.26
Worry	0.11	0.12	0.86	0.39	0.13	−0.43	0.12	−3.70	<0.001	−0.58	0.35	0.12	3.03	<0.001	0.47
Suppression	−0.33	0.12	−2.78	0.01	−0.43	−0.17	0.12	−1.45	0.15	−0.23	0.26	0.11	2.32	0.02	0.36
Sex (male)	0.06	0.25	0.23	0.82	0.05	−0.31	0.24	−1.29	0.20	−0.28	0.36	0.23	1.59	0.12	0.35
Age	0.11	0.13	0.83	0.41	0.13	−0.00	0.12	−0.02	0.99	−0.00	−0.06	0.11	−0.59	0.55	−0.09
Baseline	−0.24	0.14	−1.78	0.08	−0.28	−0.41	0.12	−3.54	<0.001	−0.55	−0.23	0.10	−2.22	0.03	−0.35
Worry × Supp.	0.35	0.12	2.78	0.01	0.43	0.12	0.11	0.98	0.33	0.15	−0.32	0.11	−2.79	0.01	−0.44

Note: Reference group, healthy controls; GAD, Generalized Anxiety Disorder; MDD, Major Depressive Disorder; SAD, Social Anxiety Disorder; d = Cohen’s d (calculated as d = t * sqrt[2/n]).

**Figure 3:**
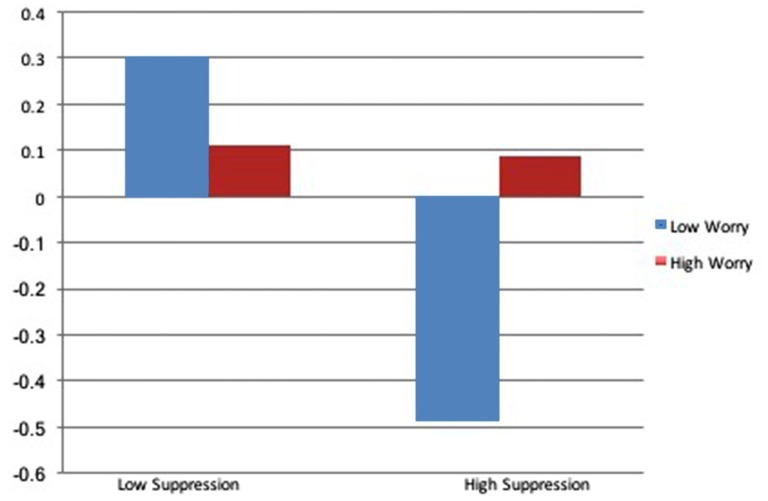
**Effect of worry on RSA throughout the course of the diagnostic interview**.

Main effects for RSA again revealed a significant effect of baseline RSA, with no additional significant predictors of RSA trajectory. The addition of the interaction between suppression and worry accounted for an additional 10% of the variance, revealing a significant interaction. For individuals with low worry severity, higher levels of suppression predicted reductions in RSA across the interview and lower levels of suppression predicted increases in RSA, however, higher levels of worry severity mitigated these results, promoting muted RSA responses across all levels of suppression. Results of RSA trajectory by worry level, as a function of emotional suppression, are depicted in Figure 3.

Worry significantly predicted decreases in PEP throughout the course of the interview, reflecting increases in SNS arousal as a function of worry severity. Baseline levels of PEP were also significant predictors of PEP trajectories, with higher levels at baseline predicting decreases across the interview. The interaction between worry and suppression was not significant.

**Figure 4:**
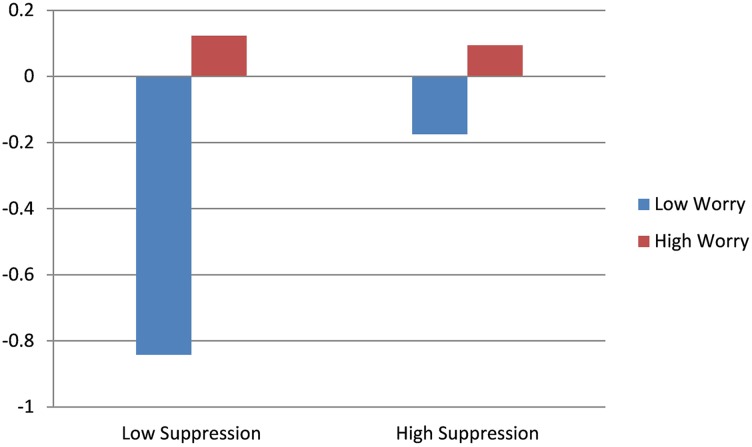
**Effect of worry on HR throughout the course of the diagnostic interview**.

Results for the main effect model of HR revealed significant effects for worry and baseline HR. Higher levels of worry predicted increases in HR throughout the course of the interview and higher baseline levels of HR predicted decreases in HR. The interaction model indicated a significant effect of suppression, whereby individuals with low levels of worry and low levels of suppression exhibited decreases in HR throughout the course of the interview. Higher levels of worry appeared to mitigate the effect of suppression on HR response, with high worry predicting non-significant change in HR, regardless of suppression level. Results of HR trajectory by worry level, as a function of emotional suppression, are depicted in Figure 4.

## Discussion

The present study investigated autonomic stress responsiveness in the SNS, PNS and HR during a semi-structured clinical interview in individuals with diagnoses of GAD, MDD, SAD and healthy controls. To our knowledge, this is the first study to directly explore autonomic stress response to a clinical diagnostic interview. Results indicated that baseline levels were significant predictors of RSA, PEP and HR trajectories, such that higher baseline levels predicted regression-to-the-mean reductions in level across the interview. Within the DSM-diagnosis models, there were no additional main or moderating predictors of PEP, although effect sizes indicated that PEP increased in healthy controls and decreased in GAD and SAD participants—indicating possible decreases in SNS arousal in controls and increases in SNS arousal in anxious participants. Suppression was a main and moderating predictor of RSA, such that greater suppression predicted reductions in RSA across all participants in the main effect model. However, the interaction model revealed that suppression predicted strong reductions in RSA for healthy controls, but that this effect was mitigated in individuals with GAD. Similarly, for HR, suppression predicted increased HR trajectories for healthy controls, with this effect mitigated for individuals with GAD. These data provide further evidence for the presence of physiological rigidity in GAD, which has been well documented in this diagnostic group (Thayer et al., 1996; Hoehn-Saric et al., 2004; Fisher et al., 2010; Fisher and Newman, 2016).

In order to examine the relative contribution of perseverative cognition to physiologic stress responsiveness, we reran analyses for RSA, PEP and HR with the transdiagnostic construct of worry in the place of clinical diagnosis. Consistent with the diagnostic models for HR and RSA, whereas trajectories for HR and RSA in those with low degrees of worry reflected increased stress responsiveness in the presence of higher suppression and decreased stress responsiveness in the absence of suppression, those with high worry exhibited flattened trajectories, regardless of the level of suppression. Also consistent with the DMS-diagnosis models, the interaction between worry and suppression on PEP was non-significant. However, contrary to the diagnostic model, worry was a significant predictor of PEP response, wherein greater worry severity predicted increased SNS arousal during the clinical interview. That worry was predictive of autonomic rigidity across clinical diagnoses suggests that the transdiagnostic feature of worry may be more predictive of a rigid, less flexible autonomic response system than the diagnosis of GAD itself, which is consistent with extant work investigate the effect of worrisome thinking on autonomic responsiveness (Borkovec and Hu, 1990; Borkovec et al., 1993; Llera and Newman, 2010).

As noted above, worry has been shown to preclude robust autonomic response to stress (Lyonfields et al., 1995), with these results extending to populations other than individuals with GAD (Borkovec and Hu, 1990). Our results suggest that engagement with worry employed those attentional and emotional systems that underpin the attenuating effect of GAD diagnosis on autonomic stress responsiveness—that is, the transdiagnostic feature alone had a stronger effect than the GAD diagnosis on measures of cardiac regulation. Though the GAD diagnosis model explained more of the variance, this finding suggests that there may be transdiagnostic features of mood and anxiety disorders, rather than specific, discrete diagnostic categories, that may be the driving physiological responsiveness to threatening or stressful demands. Future research should endeavor to investigate additional transdiagnostic features and their effects on cardiac functioning.

Results further indicated that GAD participants and individuals with high levels of self-reported worry exhibited significantly decreased PEP values over the course of the interview, indicative of greater SNS activation. Increases in RSA were also observed for low suppression GAD participants and participants with high levels of worry. Thus, the present data seem to reflect a coactivation of PNS and SNS responses—at least insofar as the 30-s epoch granularity reflected changes over time—in these individuals. The observed increases in both the PNS and SNS possibly indicate that a SNS stress response elicited a counteracting parasympathetic response. However, a larger sample of individuals with GAD and study methodology specifically aimed at disentangling these effects is likely warranted to support this hypothesis. For instance, future work, assessing the temporal structure of these data, could test the time-lagged cross-predictions between RSA and PEP time series to better understand the directionality of this effect. Additionally, there were both main and interacting effects of emotional suppression on RSA and HR trajectory during the interview for healthy controls and individuals with low levels of self-reported worry, whereby greater emotional suppression *dampened* HR and RSA response. The latter finding is consistent with existing affective literature documenting attenuating effects of emotional suppression on HR (Gross and Levenson, 1993). Again, GAD participants and those with high levels of worry were immune to these attenuating effects, indicative of physiological rigidity.

Taken together, we believe that these findings reflect both how physiologically complex a clinical interview is and how that complexity changes based on diagnostic categories and transdiagnostic features of mood and anxiety disorders. We believe that this is important for many reasons. First, a clinical interview is a ubiquitous task used by researchers and clinicians alike, oftentimes at the start of an empirical study. To understand how that may be affecting the phenomenology and physiology of an individual is crucial—for example, our findings are the first so demonstrate that suppression moderates responses in HR and RSA for healthy controls and individuals with low worry in a diagnostic interview, yet not for GAD participants and individuals with high worry—and this study is, to our knowledge, the first to examine this question. Second, our findings of reduced HR attenuation in high worriers and GAD participants throughout the interview and of the dampened moderating effect of suppression in these groups are consistent with existing literature supporting decreased autonomic flexibility in GAD (Thayer et al., 1996; Hoehn-Saric et al., 2004; Fisher et al., 2010; Fisher and Newman, 2016), while also pointing to worry being a broader preditor of this decreased flexibility, capturing variance across diagnoses. Third, these findings support current conceptualizations of emotional suppression and its effects on physiology for healthy controls and low worriers; these participants who indicated high levels of emotional suppression exhibited smaller physiological changes; that is, their physiological HR responses were diminished as a result of not fully engaging with the interview material. These findings may extend outside of the context of the interview to other situations, suggesting that healthy individuals who suppress may be predisposed to the same deleterious physical effects of autonomic rigidity as high worriers and GAD. Finally, our findings indicate distinct physiological differences across transdiagnostic features of mood and anxiety disorders. While it has been suggested that physiological responses in MDD are due to underlying comorbidities with anxiety disorders, our findings support the notion that differences may be due to shared transdiagnostic features, such as worry or suppression, rather than diagnostic comorbidities.

The present study had some key limitations, including our sample size for MDD and SAD participants being relatively small. As indicated above, trends for changes in HR with suppression were seen in GAD, MDD and SAD groups, yet only GAD was found to be significant. Future work should aim to recruit a larger sample of SAD and MDD participants. Additionally, we did not assess respiration frequency, which may have a direct influence on autonomic responses. Without controlling for respiration, it may be less clear if changes in RSA are due to changes in the parasympathetic outflow, or due to changes in respiration. Future research should endeavor to include assessments of respiration rate in order to include it is a control variable. Despite these limitations, the current study is among the first to investigate physiologic responses during a clinical diagnostic interview, and provides strong evidence for the complex nature of autonomic functioning in GAD, MDD and SAD participants in response to a diagnostic interview.

## Author Contributions

All authors contributed extensively to the work presented in this article. AJF designed the experiment. AED assembled and organized the data. AED and AJF ran the statistical analyses, analyzed output data, created the tables and figures and contributed to the writing of the manuscript.

## Conflict of Interest Statement

The authors declare that the research was conducted in the absence of any commercial or financial relationships that could be construed as a potential conflict of interest.
